# Improving the Assessment and Classification of Sick Children according to the Integrated Management of Childhood Illness (IMCI) Protocol at Sanja Primary Hospital, Northwest Ethiopia: A Pre-Post Interventional Study

**DOI:** 10.1155/2020/2501932

**Published:** 2020-10-19

**Authors:** Mohammed Abayneh, Tsegaye Gebremedhin, Endalkachew Dellie, Chalie Tadie Tsehay, Asmamaw Atnafu

**Affiliations:** ^1^Chagni primary hospital, Awi zone, Amhara region, Ethiopia; ^2^Department of Health Systems and Policy, Institute of Public Health, College of Medicine and Health Sciences, University of Gondar, Gondar, Ethiopia

## Abstract

**Background:**

A complete and consistent use of integrated management of childhood illness (IMCI) protocol is a strategic implementation that has been used to promote the accurate assessment and classifications of childhood illnesses, ensures appropriate combined treatment, strengthens the counseling of caregiver, and speeds up the referrals to decrease child mortality and morbidity. However, there is limited evidence about the complete and consistent use of IMCI protocol during the assessment and classifications of childhood illness in Ethiopia. Therefore, this intervention was implemented to improve the assessment and classifications of childhood illness according to the IMCI protocol in Sanja primary hospital, northwest Ethiopia.

**Methods:**

A pre-post interventional study was used in Sanja primary hospital from January 01 to May 30, 2019. A total of 762 (381 for pre and 381 for postintervention) children from 2 months up to 5 years of age were involved in the study. Data were collected using a structured questionnaire prepared from the IMCI guideline, and a facility checklist was used. A five-month in-service training, weekly supportive supervision, daily morning session, and availing essential drugs and materials were done. Both the descriptive statistics and independent *t*-test were done. In the independent *t*-test, a *p* value of <0.05 and a mean difference with 95% CI were used to declare the significance of the interventions.

**Results:**

The findings revealed that the overall completeness of the assessment was improved from 37.8 to 79.8% (mean difference: 0.17; 95% CI: 0.10-0.22), consistency of assessment with classification from 47.5 to 76.9% (mean difference: 0.34; 95% CI: 0.27-0.39), classification with treatment from 42.3 to 75.4% (mean difference: 0.35; 95% CI: 0.28-0.47), and classification with follow-up from 32.8 to 73.0% (mean difference: 0.36; 95% CI: 0.29-0.42).

**Conclusion:**

The intervention has a significant improvement in the assessment and classification of childhood illness according to the IMCI protocol. Therefore, steps must be taken to ensure high quality of training, adequate supervision including the observation of health workers managing sick children during supervisory visits, and a constant supply of essential drugs and job aids for successful implementation of IMCI in the hospital and also to other facilities.

## 1. Introduction

The integrated management of childhood illness (IMCI) is a globally proven evidence-based intervention designed to improve child survival and is being implemented worldwide in countries with a high burden of child mortality [1, 2]. The guidelines for the IMCI were introduced by the World Health Organization (WHO) and United Nations International Children's Emergency Fund (UNICEF) in the mid-1990s to enable health care workers to diagnose and treat a sick child in locations with limited access to laboratories and medical equipment targeting on major causes of under-five mortality [3–5]. Every year, 8 million children in developing countries die of preventable disease before they reach their fifth birthday; many of them die during the first year of life. Eight out of ten of these deaths are due to child and neonatal conditions, acute respiratory infection (mostly pneumonia), diarrhea (including dysentery), malaria or severe malnutrition, or a combination of these conditions. The majority (70%) of deaths and 80-90% of illness are due to the above childhood disease [5, 6].

The complete and consistent use of IMCI protocol in Ethiopia was introduced since 1997 and is considered as an evidence-based intervention for accurate identification of childhood illnesses, ensures appropriate combined treatment of all major diseases, strengthens the counseling of caregiver, and speeds up the referral of severely ill children. In the home setting, it promotes appropriate care-seeking behaviors, improved nutrition, and preventative care, and the correct implementation of prescribed care [5, 7]. The guidelines are presented in a chart booklet that contains step-by-step instructions on how to assess and treat sick children [6].

After IMCI implementation started in Ethiopia, under-five mortality is significantly reduced from 92.8/1000 live births in 2008 to 55.2/1000 live births in 2018 [8]. Despite these, children less than five years still die in large numbers [9]. Improving the quality of care in child health services by full use of the IMCI protocol is essential for further substantial reductions in the under-five children mortality [10]. Using IMCI has become a primary child survival intervention in almost all countries in African including Ethiopia by creating a better opportunity to scale up children's health interventions. Incorporating the algorithms in the IMCI protocol and strengthening the components of the strategy related to the health system and community will directly improve child health. It functions best when families and communities are linked to the first facility level, which in turn links well to the referral level. IMCI combines prevention and care, focusing on the child and not only on the individual's diseases [11, 12]. Despite the above advantages' completeness and consistent use of IMCI protocol, registration and algorithm adherence are challenged with different contextual factors in Ethiopia specifically in Sanja primary hospital. Some of the contributing factors are different intrinsic and extrinsic motivating factors of professionals, reduced availability of supplies and equipment, shortage of essential drugs in the hospital, staff turnover, limited supportive supervision, poor documentation practices, high patient load, long waiting time, limited professionals' capacity building training, lack of periodic performance evaluation, and other related problems that may be attributed to unmatched child health problem identification and treatment and poor follow-up arrangement that leads to high preventable deaths [6, 13, 14]. Moreover, sociodemographic factors such as income, marital status residency, and educational level of parents may contribute to IMCI implementation in health facilities [15]. However, there are little pieces of evidence about the completeness and consistency of child health assessment with disease classification and its management according to the IMCI protocol in Ethiopia, particularly in primary level health facilities. Even most of the studies done are cross-sectional studies [16, 17]. Hence, to reduce these preventable child mortalities, improving the proper utilization of IMCI protocols with the support of scientific evidence is recommended. Therefore, the objectives of interventions were to improve the assessment and classification of childhood illness according to the IMCI protocol in Sanja primary hospital, northwest Ethiopia, through training, frequent supportive supervision, and proper placement of health care professionals. Furthermore, these interventions may improve the understanding of barriers and facilitators to IMCI implementation in the hospital, describing how the quality improvement team, hospital management team, and researchers collaboratively develop strategies to improve the adherence of IMCI implementation and ascertaining whether the strategies present a feasible and effective alternative for strengthening IMCI implementation in the hospital and similar settings.

## 2. Methods and Materials

### 2.1. Study Design and Settings

A pre-post interventional study was employed to improve the assessment and classification of childhood illness using the IMCI protocol in Sanja primary hospital from October 01, 2018 to May 30, 2019. Sanja primary hospital is located in northwest Ethiopia, which is 230 and 60 kilometers far from Bahirdar (city-state of Amhara region) and Gondar town, respectively. The hospital provides outpatient, adult and child, inpatient, neonatal, and other diagnostic and laboratory services for around 167,587 catchment population and has 148 human resources in all categories. The average daily patient visit of the under-five OPD was estimated to be 39 cases per day. The district where the hospital found is very hot for most of the season. According to the 2017 hospital annual report, the top three leading causes of outpatient visits in the hospital were as follows: malaria, pneumonia, and urinary tract infection. Particular to the under-five OPD, pneumonia, malaria, and diarrheal disease were the top three leading causes of diseases [18]. This interventional study involves the participation of many actors. The hospital senior management team and quality improvement team are involved in the development of the intervention, all under-five unit staff applied the intervention, and the research team involved in the evaluation of the intervention. All children aged 2 months to 5 years who were treated from October to December 2018 for preintervention and March to May 2019 for postintervention in the hospital were the source population for the study, whereas all children between 2 months and 5 years of age who were treated and registered on the IMCI protocol from October to December 2018 for preintervention and January to May 2019 for postintervention in Sanja primary hospital were the study population. The cadres use the IMCI guideline to register and manage all sick children who visit the under-five OPD either by referral from lower-level facilities or who come to seek care at the facility without referral [7, 19]. The IMCI guideline for the management of sick children was not amended during the intervention period, and there are no standard criteria about the number of cases the cadres can see in a day. If the sick children are seriously ill, the cadres working at the under-five OPD consult with the pediatrician and refer them to the hospital's pediatric emergency unit for further management. IMCI classifications are action-oriented and allow a health care provider to determine whether a child should be referred to another health facility as a matter of urgency, whether the child can be treated at the first-tier facility (e.g., with oral antibiotics, antimalarial, and ORS), or whether the child can be managed safely at home.

### 2.2. Interventions

Initially, incomplete and inconsistence implementation of the IMCI protocol/booklet chart is prioritized as the most critical problem that required intervention from the many problems identified by the multidisciplinary team (e.g., a shortage of laboratory reagents, long OPD waiting time, low bed occupancy rate, incomplete use of IMCI protocol/booklet chart, low postnatal care follow-up, high emergency referral rate, and poor infection prevention practice).

The preintervention data assessment performed using a structured checklist/audit tool by reviewing 381 under-five IMCI protocol registrations on 18 IMCI protocol formats, and the completeness and consistency of each component showed IMCI completeness 37.8%, consistence of classification of disease 47.5%, consistency treatment 42.3%, and consistence of follow-up 34.1% which is low against the standard set by WHO (68%) for completeness and follow-up.

**Figure 1:**
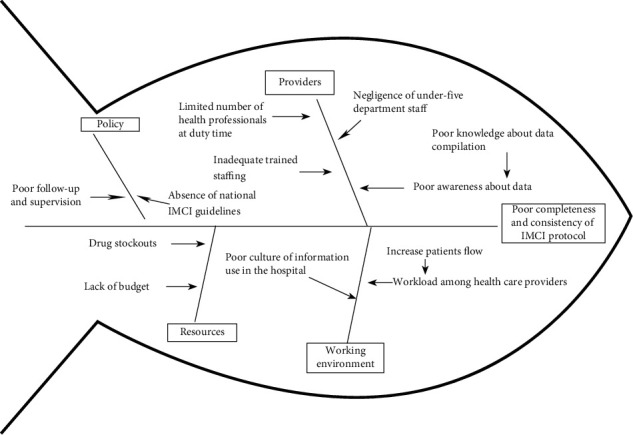
Fishbone diagram analysis for the root causes of poor completeness and consistency of IMCI protocol in Sanja primary hospital, northwest Ethiopia, 2019.

Then interventions for improving the consistency and completeness of IMCI protocol in the hospital were identified through a multidisciplinary team participatory approach. The multidisciplinary team was composed of the senior hospital management team, under-five clinic coordinator (chief clinical nurse), physician, hospital quality officer, plan and program officer, and health management information system (HMIS) officer. The team initially identified the possible root causes for the low consistency and completeness of the IMCI protocol using the fishbone root cause analysis after conducting desk review, consulting senior pediatrician and quality officer, findings from routine observation, necessary document review in the under-five clinics, and reviewing empirical evidence. The major causes for the low consistency and completeness of IMCI protocol were categorized under people, policy, equipment, and environment themes, and the possible root causes were subsequently explored from those major causes by using fishbone analysis (Figure 1).

For the identified root causes, the possible interventions were determined for each root cause through the assumptions of the following: the intervention will be addressed the root cause directly, in line with the aim of the intervention, the visibility of its indicators for measurement, and its possibility of increasing professional adherence to the IMCI guideline. Then, those interventions were prioritized using the intervention prioritization criteria to select the best interventions. The criteria used for the comparison and selection of best interventions were effectiveness/impact, time, feasibility, and cost. Each criterion was measured by using a five-point Likert scale (from one to five), and the highest scores were considered as the best intervention to improve the low consistency and completeness of the IMCI protocol (Table 1).

Accordingly, the best interventions were providing intensive training to health professionals working on under-five clinics, proper assignment of the health professionals, improving commitment of the health professionals, increased number of U5 OPD, availing of IMCI registration, follow-up absenteeism in duty time, availing of IMCI guideline, regular and frequent supportive supervision, and availing the essential drugs and medicines for under-five clinics. The overall interventions were described and presented using the logical framework matrix (Table 2).

### 2.3. Implementations

A total of 12 health care providers working in the under-five OPD, and three supervisors were received 05 days of IMCI clinical management training to fill their knowledge and skill gaps and to improve their motivation and attitude for the use of data. The training was given in collaboration with the University of Gondar and Amhara National Regional Health Bureau at the University of Gondar comprehensive specialized hospital training hall by expertise who have attended the Training of Trainers (TOT). Pre- and posttest were undertaken to ascertain whether the trainees had attained the required knowledge and competencies or not. Besides, on the last day of the training, the trainees had a practical session on actual patients under the supervision of the trainers to verify their competency. Besides, the hospital avails all the necessary IMCI protocol formats and materials. Of the 12 trainees, 4 were general practitioner doctors, 2 were BSc degree holder health officers, 2 were BSc degree holder nurses, and 4 were diploma holder nurses. Regarding their work experience, 8 (66.7%) had worked 2 to 5 years. After the training, the discussion was held with the hospital senior management team to give full support for the under-five department.

In early March 2019, postimplementation of the IMCI case management protocol was started. Health facility recording and reporting systems were updated every week to improve information exchange on sick child management and the use of drugs in the under-five OPD. A facility-level drug tracking and reporting system were introduced that is in line with the regional health bureau. Structured formats for providing weekly reports were availed at the under-five OPDs.

The supervisors had been visiting the interventions without prior notice every week. Using a specially designed supervisory checklist, the supervisor reviewed the patient register to check for completeness and consistency of records and to determine overall caseloads. The supervisors also were observing the IMCI trained providers in the management of at least five sick children and providing immediate written feedback and reinforcement. The duration of each supervisory visit ranged between one to one and a half hours. But the weekly supervisory visit schedule was rarely missed. The project investigator, who was visiting the under-five once a month, was also visiting each of the availability of aid formats twice a month to collect information regarding facility utilization. However, observation of case management was not included in these visits. The intervention has been implemented through a full monitoring and evaluation session, and the overall implementation lasted from January to May 2019 for five months' duration.

### 2.4. Outcome Measures

*Consistency of assessment with classification*: the number of malaria, pneumonia, and diarrhea case diagnoses in line with the IMCI protocol in Sanja primary hospital by the end of May 2019.

*Consistency of classification with treatment*: the number of malaria, pneumonia, and diarrhea cases treated in line with the IMCI protocol in Sanja primary hospital by the end of May 2019.

*Consistency of classification with follow*-*up*: the number of malaria, pneumonia, and diarrhea cases correctly appointed for follow-up in line with the IMCI protocol in Sanja primary hospital by the end of May 2019.

*Completeness of IMCI protocol*: the number of children treated by using complete IMCI protocol in Sanja primary hospital by the end of May 2019.

### 2.5. Secondary Outcomes (Outcome Measures)

The number of trained physicians, health officers, and nurses on the uses of IMCI protocol for the assessment and classification of childhood illness and the number of supportive supervisions conducted regularly and frequently per week in under-five OPD in Sanja primary hospital by the end of May 2019 were the process measures.

### 2.6. Operational Definitions

Consistency of the IMCI protocol was measured by consistency of assessment with classification+consistency of classification with treatment+consistencies of classification with follow-up.

*Consistency of assessment with classification*: cases that are classified based on their assessment appropriately by using the IMCI protocol.

*Consistency of classification with treatment*: cases that are treated based on their classification appropriately by using IMCI protocol.

*Consistency of classification with follow-up*: cases that have follow-up/appointment given based on IMCI protocol.

*Completeness of IMCI protocol*: cases that are checked by all 18 components of IMCI protocol.

*Danger sign assessment is said to be completed*: if a health worker asked the mother about child feeding, vomiting everything, conversion, and checked the presence of lethargic or unconsciousness. If one of these was missed, it was said to be an incomplete assessment.

*Assessment for cough or difficulty of birthing is said to be completed*: if a health worker checked the duration of coughing, checked respiration, checked chest indrawing, and wheezing. If one of these is missed it was said to be incomplete.

*Assessment for diarrhea status is said to be complete*: if a health worker checked the duration of diarrhea, the presence of blood in the stool, checked the presence of sunken eye, checked the response to drink and determining whether the child is not able to drink or drinking poorly or drinking eagerly, and checked skin pinch and determining whether it is intact, slow, or very slow. If one of the above is missed it was said to be incomplete.

*Assessment for fever status is said to be completed*: if the child's temperature was measured and the blood film was done.

### 2.7. Population and Sampling Procedures

The sample size was determined for chart review (under-five child individual folder) the assessment of the preintervention and postintervention results using a single population proportion formula with the assumption proportions of the completeness of IMCI was 54.2% taken from a study conducted in Shire government hospitals, Ethiopia [20], 95% confidence level (CI), and a margin of error (5%). Then, putting the value in the formula, it gives the final sample of 381. So, 381 under-five charts were reviewed for preintervention and postintervention assessments. A systematic random sampling technique was applied to get the calculated samples from the IMCI registers using their medical record number. The first study participant (a medical record number) was selected from the IMCI registers randomly by using the lottery method, and the rest of the participants were selected by using the skipping intervals until the required sample size was maintained.

### 2.8. Data Collection Tools and Procedures

The data were collected by reviewing registration using a format similar to IMCI protocol which has 18 items/domains: assessing ill child, classifying for a sick child, treating with medication, weight, height, temperature, general danger sign, cough/difficulty of birthing status, diarrheal status, fever, ear problem, anemia, immunization status, HIV/AIDS status, TB screening, feeding conduction, nutrition status counseling caregivers, and need for referral [7]. Three BSc nurses for data collection and one health officer for supervision who were working out of Sanja primary hospital were recruited. Data collectors and the supervisor were oriented and practiced for three days at Sanja primary hospital about a structured checklist/questioner, on data review techniques, the purpose of the study, and the importance of privacy and discipline. Data quality control orientation is given to data collectors and the supervisor, and completed forms were reviewed and edited weekly by supervisors.

### 2.9. Data Quality Control

Before the beginning of data collection, data collectors were given a full course of training regarding the basic techniques of data collection procedures and how to collect quality data. The principal investigator and supervisors were making a day to day on-site supervision during the whole period of the data collection process for both pre- and postinterventions. At the end of each day, the checklists were checked for completeness, accuracy, and consistency.

### 2.10. Data Management and Analysis

The completed data were coded, cleaned, and entered into EPI-Data version 3.1 and exported to the SPSS version 23 software for analysis. Descriptive statistics were done and presented using appropriate descriptive measures such as text, tables, frequencies, and percentages. Finally, the independent *t*-test was used for comparing pre- and postintervention, and a *p* value of <0.05 and a 95% confidence level was used to declare the significance of the intervention.

### 2.11. Ethics Approval and Consent to Participate

Ethical approval was obtained from the Ethical Review Committee of the Institute of Public Health, the University of Gondar (Ref. No.: IPH8375/06/19), and a supporting letter was obtained from Sanja primary hospital. Written consent was obtained after explaining the purpose and importance of the intervention before the problem identification phases from the health care providers at the hospital. Approval was obtained from the hospital management board after reviewing the risk and benefits of the study, and then permission was taken from the outpatient department coordinator. The investigators explained the participants about the procedures, risks, and benefits of the study. Additionally, investigators also ensured that participants understood the information they provided to decide whether they want to participate; then, verbal consent was obtained from each study participant and from a parent or guardian for participants under 16 years old study participants to ensure their voluntariness to participate in the study. The data were obtained after full revision of the under-five registrations and desk discussion with key personnel, and then confidentiality was maintained by excluding the names and other identifiers.

## 3. Result

### 3.1. Completeness of Assessment and Classification of Childhood Illness Using IMCI Protocol

A total of 762 (381 preintervention and 381 postintervention) children treated in the under-five OPD of IMCI registration were reviewed. The majority (51.3% pre and 53.5% post) were females, and 46% at preintervention and 42% at postintervention were in the age group of 2-4 years. Most of the children (33% at pre and 37% at postintervention) came with the chief complaint of dry cough and fever. All the required formats were 100% available, and there was no missed patient's registration. Out of 381 cases, 293 (76.9%) were given classification, and for 287 (75.3%) cases, treatment was prescribed. Additionally, 278 (73%) cases have given appointments. Out of classified cases, 117 (39.6%) were pneumonia cases, 85 (29.0%) were malaria, and 73 (24.9%) were diarrheal diseases, and the rest 19(6%) were other diseases. Nearly eighty percent of the treatment were completed as per the IMCI protocol (Table 3).

### 3.2. Consistency of Assessment and Classification of Childhood Illness Using IMCI Protocol

With related to the classification of the top three child diseases, 85.3, 87.7, and 95.3% pneumonia, diarrhea, and malaria cases were correctly classified in line with the IMCI protocol, respectively. Concerning treatment in line with the IMCI protocol, 74.1, 78.1, and 94.1% of pneumonia, diarrhea, and malaria cases were correctly treated as per the protocol, respectively. Moreover, 68.9, 64.4, and 74.2% of pneumonia, diarrhea, and malaria cases were appointed in line with IMCI protocol, respectively (Table 4).

### 3.3. Primary and Secondary Outcomes

**Figure 2:**
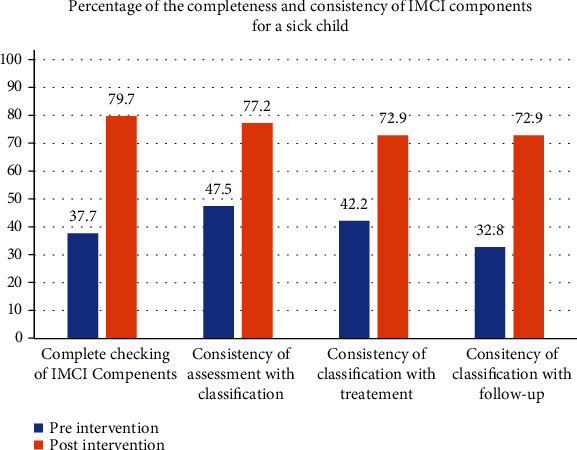
Consistency and completeness of the assessment and classification of childhood illness using IMCI protocol in Sanja primary hospital, northwest Ethiopia, 2019.

In the primary outcome, there was a change of the completeness of IMCI protocol from 37.8 to 79.8%, followed by the consistency of classification with follow-up in line with the IMCI protocol from 32.8 to 73.0%. In the secondary outcomes, the number of trained physicians and nurses increased from 8 to 75%, an additional health care profession was recruited, and supportive supervision was conducted weekly in the postintervention (Figure 2).

### 3.4. Testing of the Interventions

The significance of the intervention was checked using the independent *t*-test. The significance of the intervention was measured for each IMCI protocol component and the overall completeness and consistency. Accordingly, there were tremendous positive changes in the assessment of other problem for a sick child (mean difference: 0.44; 95% CI: 0.37-0.52) and lower in the assessment of fever (mean difference: 0.22; 95% CI: 0.16-0.28). The intervention has positive changes (mean difference: 0.17; 95% CI: 0.10-0.22) on the overall completeness of the assessment and classification of childhood illness using the IMCI protocol (Table 5).

Moreover, the intervention has a higher significance changes on the consistency of classification with follow-up (mean difference: 0.36; 95% CI: 0.29-0.42) according to the IMCI protocol positive changes (mean difference: 0.17; 95% CI: 0.10-0.22) on the overall completeness of the assessment and classification of childhood illness using the IMCI protocol (Table 6).

## 4. Discussion

The overall consistency and completeness of IMCI protocol in this pre-post interventional study were 45.27 and 59.7% before the intervention and 83.63 and 80.4% after the interventions, respectively. Many scholars agreed that MCI guideline is the best tool for the accurate management of under five-year children. It can significantly avoid discrepancies among health institutions and health professionals in the management of childhood illness. This facility-based pre-post interventional study was tried to measure the level of improvement of assessment and classification of childhood illness using the IMCI protocol. The study findings suggest that with good quality training and regular supportive supervision, completeness and consistency of the use of IMCI protocol can be sustained, but this research showed at preintervention health professionals were classifying and treating cases incorrectly compared to postintervention. The findings are in line with a multicountry study finding that shows that the IMCI guideline has a great effect on the reduction of under-five morbidity and mortality with the health professional inconsistency usage of classification and treatment [3, 4].

Our study findings showed that the proportion completeness of IMCI implementation was 59.7% at preintervention and 80.4% at postintervention. The preintervention showed below the standard level established (68%) by WHO and UNICEF [17], and the postintervention was above the standard, and this study finding is quite higher than that of the study conducted in China which was 42.3% [21]. This difference may be related to the negligence of health professionals, lack of training, infrequent supervision, and maybe also related to the difference in the qualification of health professionals.

Our interventional study also showed that consistency in assessing and classifying sick children was 49.4 and 82.5% for pre- and postinterventions, respectively, which was better than that of a study conducted in the Shire, northern Ethiopia, which showed that only 62.8% were correctly classified [20]. Moreover, this study also indicates higher consistency in assessing and classifying sick children from the research conducted in China, which showed that only 43.8% [21] and Tanzania 28.4% [22].

The study finding showed that the assessment of each component of the IMCI protocol for a sick child had increased form the pre to postintervention. The more enormous changes were observed in the assessment of HIV/AIDS status (37.8 to 79.9%) and giving of appointment (32.8 to 73.0%), and lower results were observed in the assessment of ear problem (60.3 to 87.5%) and fever status (63.0 to 85.3%). Those findings were better than the studies conducted in China which showed a sick child who was checked for the weight (4.9%), body temperature (60.3%), danger signs (2.87%), cough, diarrhea, and fever (10.9%), height (0.3%), vaccination (4.6%), feeding problem (5.21%), and other problems (29.95%) [21]; in Shire which showed a sick child who was checked for danger signs (40%), vaccination status (22.5%), and HIV status (15%) [20], and the WHO assessment of IMCI showed that a sick child was checked for pneumonia (34%), diarrhea (18.6%), and cough or difficulty of birthing (39.8%) [7]. The possible reasons for this difference might be the difference in study setup and study design; some of the studies were a cross-sectional study like in China and the WHO assessment.

In our study, the consistency of treatment of childhood illness with the classification of disease at the pre- and postintervention was 48.9 and 85.2%, respectively. The finding was higher than a study conducted in Benin, which showed about 63.6% [23], and in the Shire, northern Ethiopia, about 42.7% [20] of the children treated according to IMNCI guideline. The possible difference might be the difference in the performance of individual health workers and skills to manage a sick child using the IMCI guideline. The discrepancy is mostly due to the difference of individual approaches to adhering to the using IMCI. Some studies noted a decline in the performance and adherence rates of health professionals to use IMCI protocol to treat childhood illness is depending on the time since the last IMCI training or retraining was taken [24, 25], whereas others could not confirm these results, what makes health professionals did not correctly implement the IMCI protocol in the health facilities [26, 27]. But a study conducted in Tanzania shows that there was an increment of use of the IMNCI guideline for the treatment of a sick child. This difference could be due to the focus of the policy, intensive training, and frequent supportive supervision [22]. Moreover, this study finding is comparable with a study conducted in India [28].

The study shows that there were higher changes from 37.47 to 82.3% in the appointment or follow-up of a sick child by the intervention which is higher compared to the WHO recommendation (68%) [7], Brazil (59%) [29], and in the Shire (24.7%) [20] findings. Likewise, a study conducted in Bangladesh shows that children were fully assessed or correctly treated but there was a major problem in advising and follow-up [24].

Our intervention demonstrates that in-service training on IMCI case management was given for 58.33% health professionals working in the under-five OPD accompanied by weekly supervision that includes observation of health worker performance, individual feedback, availability of essential medicines, and formats to improve the quality of child health care in the hospital setting was implemented. The findings were compared with the WHO recommendation in which at least 60% of health care workers seeing a sick child in the health facility should be trained on IMCI [17]. There is evidence that supervision can improve performance, systems for monitoring are usually available, and supervisors are often the only human link between the first level health workers and the health system. The quality of supervision and the amount of time spent by supervisors with health workers are the main challenges for proper supervision.

### 4.1. Strength and Limitation of the Study

Since this study was an interventional and that stayed for five months, it can show the real effect of the intervention on the assessment and classification of the childhood illness according to the IMCI compared to other interventional studies that stay for not more than three months. Besides, the interventions were provided by trained and more experienced health care providers that might show the actual effects of the interventions. Moreover, the intervention was used as a representative sample for the IMCI protocol for generalizations to other service areas and hospitals. The limitations are as follows: the study used a specific study area that might not include other factors like infrastructure variation across facilities that can affect the intervention, so it will be better to add more facilities to see the facility difference by the interventions. To minimize those differences, at the baseline, we identified the possible root causes using a multidisciplinary team. The use of secondary data that might be difficult to locate the specific professional category and training status at the baseline, but for the postintervention, we gave training and supervision almost for all health workers working at under-five OPD.

The absence of a comparison group will make it difficult to know whether the improvements observed are because the staffs of the facility know that they are being evaluated and other secular trends or whether it is a true intervention effect. The data sources may be biased because the people whose performance is being evaluated are also those that record data in the registers.

The improvement in performance might be an artifact of improved record-keeping and documentation of service delivery and not better IMCI implementation. The service providers that cared for the sick children in the preintervention group might not be the same people that cared for them in the postintervention group.

The methodology does not allow us to assess whether the IMCI steps performed were done correctly, rather we can only know whether they were carried out or not and whether succeeding steps are logical course to follow based on the step that preceded them. To assess the acceptability, perception of health workers, and sustainability of the intervention, qualitative data would be required that this study could not address.

## 5. Conclusions

The study findings suggested that with good quality training and consistent supportive supervision, adequate performance on the assessment and classifications of the childhood illness among IMCI trained service providers can be sustained to have a more tremendous effect. Besides, training in IMCI improves the performance of health teams, the understanding, and practices of caregivers, especially concerning the advice to the caregiver. Moreover, after the intervention, the under-five departments were visited weekly by trained supervisors who spent a substantial amount of time observing the health workers as per the IMCI protocol that can sustain a good quality of care for sick children. After the intervention, the IMCI program is regarded positively by all health care workers in the hospital, and they are optimistic about a full implementation of IMCI if there is a collaboration of supervisors, IMCI focal person, hospital management, and other stakeholders interested in the child health. Therefore, steps must be taken to ensure high quality of training, adequate supervision including the observation of health workers managing sick children during supervisory visits, and a constant supply of essential drugs and job aids for successful implementation of IMCI in Sanja primary hospital.

## Figures and Tables

**Table 1:** Prioritization matrix to select possible intervention to solve incompleteness and inconsistency of use of IMCI protocol in Sanja primary hospital, northwest Ethiopia, 2019.

List of interventions	Prioritization criteria					
	Cost	Impact	Time	Feasibility	Total	Rank
Provision of training to health professional	5	5	4	4	18	1
Proper placement of health professionals	5	4	4	4	17	2
Improving commitment of the health professionals	5	3	3	3	14	5
Increased number of U5 OPD	4	3	3	3	12	7
Availing of IMCI registration	5	3	2	2	11	8
Follow-up of duty hour gap of professionals	4	4	4	3	15	4
Availing of IMCI guideline	3	3	4	3	13	6
Regular and frequent supportive supervision	4	4	5	3	16	3
Avail all the necessary drugs and medical supplies	2	4	2	3	11	8

IMCI: integrated management of childhood illness; U5: under five; OPD: outpatient department.

**Table 2:** Log frame matrix for improving the assessment and classifications of childhood illness according to the IMCI protocol in Sanja primary hospital, northwest Ethiopia, 2019.

Description of the interventions	OVI^∗^	Data sources	Assumptions
Goal: Improving the quality of health service under 5 OPD	(i) No. of morbidity in the hospital	(i) HMIS reports (ii) KPI reports (iii) EHSTG report	(i) If the program is functional (ii) If trained professional work in line with IMCI protocol (iii) If enough nurse in under-five OPD (iv) If all information in the IMCI protocol is filed completely
Objective: Improving the completeness and consistency of IMCI protocol	(i) No. of children who are safer from repeated infection of the same disease	(i) IMCI protocol registration (ii) Individual folder	
Strategies: (i) Regular and frequent supportive supervision (ii) Training to health worker (iii) Proper placement of health workers	(i) No. of supervision per week (ii) No. of IMCI trained-health worker (iii) No. of health workers who work in under-five OPD	(i) Observing program logbook (ii) Human resource department training register (iii) Hospital monthly professional placement logbook	
Activities (i) Select nurse for supervision (ii) Give orientation on how to supervise with programmed and frequent supportive supervision (iii) Giving 5 days training for 20 health professionals on IMCI protocol implementation (iv) Purchasing and preparing the same training materials (v) Conducting a baseline assessment on IMCI training (vi) Preparation of training modules for training (vii) Practical pre-post testing of the trainee on U5 OPD on IMCI training (viii) Assign the nurse in class depending on information get (ix) Give the program to responsible body and post on class and follow (x) Monitoring and evaluation of the result			

EHSTG: Ethiopian Hospitals Standards Treatment Guideline; HMIS: health management information system; IMCI: integrated management of childhood illness; KPI: key performance indicators; OPD: outpatient department; OVI: objectively verifiable indicators; U5: under five.

**Table 3:** Completeness of the IMCI protocol component for a sick child in Sanja primary hospital, northwest Ethiopia, May 2019.

IMCI components' checked for a sick child	Completeness of the assessment and classification of the childhood illness				*χ* ^2^
	Preintervention (*n* = 381)		Postintervention (*n* = 381)		
	Complete *n* (%)	Incomplete *n* (%)	Complete *n* (%)	Incomplete *n* (%)	
Height	162 (42.5)	219 (57.5)	304 (79.8)	77 (20.2)	111.4
Weight	228 (59.8)	153 (40.2)	309 (81.1)	72 (18.9)	41.3
Temperature	209 (54.9)	172 (45.1)	311 (81.6)	70 (18.4)	62.9
Danger sign	220 (57.7)	161 (62.3)	322 (84.5)	59 (15.5)	66.5
Cough/difficulty of birthing	198 (52.0)	183 (48.0)	333 (87.4)	48 (12.6)	113.2
Diarrheal status	221 (58.0)	160 (42.0)	331 (86.9)	50 (13.1)	79.5
Fever status	241 (63.0)	140 (37.0)	325 (85.3)	56 (14.7)	48.46
Ear problem	232 (60.3)	149 (39.7)	334 (87.5)	47 (12.5)	71.46
Anemia status	224 (58.8)	157 (41.2)	325 (85.3)	56 (14.7)	66.4
Immunization status	183 (48.0)	198 (52.0)	327 (85.8)	54 (14.2)	122.9
HIV/AIDS status	144 (37.8)	237 (62.2)	306 (79.9)	75 (20.1)	142.4
TB status	194 (50.9)	187 (49.1)	320 (84.0)	61 (16.0)	94.9
Feeding problem.	179 (47.0)	202 (53.0)	314 (82.4)	67 (17.6)	104.7
Nutrition status	197 (50.9)	184 (49.1)	327 (85.8)	54 (14.2)	103.2
Give classification of the disease	181 (47.5)	200 (52.5)	293 (76.9)	88 (23.1)	70
Prescribe treatment for the disease	161 (42.3)	220 (57.7)	287 (75.4)	94 (24.6)	85.9
Give appointment	125 (32.8)	256 (67.2)	278 (73.0)	103 (27.0)	123.2
Other problem	226 (59.3)	155 (40.7)	297 (78.0)	84 (22.0)	30.7
Completeness of full IMCI protocol	144 (37.8)	237 (62.2)	304 (79.8)	77 (20.2)	138.6

*χ* ^2^: chi-square.

**Table 4:** Consistency of the assessment and classification of common childhood illness using the IMCI protocol in Sanja primary hospital, northwest Ethiopia, 2019.

Disease type	Consistency of IMCI protocol											
	Assessment with classification		Treatment with classification								Appointment with classification	
	Pre, *n* (%)	Post, *n* (%)	Pre, *n* (%)				Post, *n* (%)				Pre, *n* (%)	Post, n (%)
			Treated	Not treated	Consistent Rx. with classification	Treated but not consistent with the classification	Treated	Not treated	Consistent Rx. with classification	Treated but not consistent with the classification		
Pneumonia (117)	67 (57.3)	99 (85.3)	111 (95)	6 (5)	60 (54.1)	51 (54.9)	115 (98.3)	2 (1.7)	86 (74.1)	29 (25.9)	49 (42.2)	80 (68.9)
Diarrhea (73)	44 (60.2)	64 (87.6)	71 (97.3)	2 (2.7)	39 (54.2)	32 (45.8)	73 (100)	0 (0)	57 (78.1)	16 (21.9)	31 (42.0)	47 (64.4)
Malaria (85)	54(63.6)	81 (95.2)	82 (96.5)	3 (3.5)	48 (58.4)	34 (41.6)	84 (98.8)	1 (1.2)	79 (94.1)	5 (5.9)	30 (35.3)	63 (74.2)

Rx.: treatment.

**Table 5:** Independent *t*-test of the intervention on the completeness of IMCI components in Sanja primary hospital, northwest Ethiopia, 2019.

Variables	df	Mean difference with 95% CI
Check child height	760	0.37 (0.30-0.43)^∗^
Check child weight	760	0.27 (0.14-0.27)^∗^
Check child temperature	760	0.22 (0.20-0.33)^∗^
Check child danger sign	760	0.27 (0.20-0.32)^∗^
Check child cough/difficulty of birthing	760	0.35 (0.29-0.41)^∗^
Check child diarrheal status	760	0.29 (0.22-0.34)^∗^
Check child fever status	760	0.22 (0.16-0.28)^∗^
Check child ear problem	760	0.26 (0.20-0.32)^∗^
Check child anemia status	760	0.26 (0.20-0.32)^∗^
Check child immunization status	760	0.38 (0.31-0.43)^∗^
Check child HIV/AIDS status	760	0.42 (0.35-0.43)^∗^
Check child TB status	760	0.33 (0.26-0.39)^∗^
Check the child feeding problem.	760	0.35 (0.29-0.41)^∗^
Check child nutrition status	760	0.34 (0.28-0.40)^∗^
Check child correct classification of disease	760	0.34 (0.27-0.39)^∗^
Check child correct treatment of disease	760	0.35 (0.28-0.41)^∗^
Check child correct appointment of disease	760	0.36 (0.29-0.42)^∗^
Check child other problem	760	0.44 (0.37-0.52)^∗^
Check full components of the IMCI protocol	760	0.17 (0.10-0.22)^∗^

df: the degree of freedom (*n* − 2); ^∗^statistically significant at *p* value <0.001.

**Table 6:** Independent *t*-test of the intervention on the consistency of the assessment and classification of common childhood illness using the IMCI protocol in Sanja primary hospital, northwest Ethiopia, 2019.

Consistency	df	Mean difference with 95% CI
Consistency of assessment with classification	760	0.34 (0.27-0.39)^∗^
Consistency of classification with treatment	760	0.35 (0.28-0.41)^∗^
Consistency of classification with follow-up	760	0.36 (0.29-0.42)^∗^

df: degree of freedom (*n* − 2); ^∗^statistically significant at *p* value <0.001.

## Data Availability

Data will be available upon reasonable request from the corresponding author.

## Abbreviations

HMIS: Health management information system
IMCI: Integrated management of childhood illness
OPD: Outpatient department
SPSS: Statistical package for social sciences
U5: Under-five years
UNICEF: United Nations International Children's Emergency Fund
WHO: World Health Organization.
